# Food protein‐induced enterocolitis syndrome: A large French multicentric experience

**DOI:** 10.1002/clt2.12112

**Published:** 2022-02-17

**Authors:** Anaïs Lemoine, Anne‐Sophie Colas, Sébastien Le, Christophe Delacourt, Patrick Tounian, Guillaume Lezmi

**Affiliations:** ^1^ Department of Pediatric Nutrition and Gastroenterology Trousseau Hospital AP‐HP Sorbonne Université Paris France; ^2^ Department of Pediatric Pneumology and Allergology Necker‐Enfants Malades Hospital AP‐HP Université Paris Descartes Paris France; ^3^ Pediatric Emergency Unit Trousseau Hospital AP‐HP Paris France; ^4^ Department of Pediatric and Emergency Unit Louis Mourier Hospital AP‐HP Université de Paris Paris France

**Keywords:** children, FPIES, France, severity, tolerance

## Abstract

**Background:**

Food protein‐induced enterocolitis syndrome (FPIES) is a non‐IgE‐mediated food allergy, with potential dehydration secondary to vomiting. Differences exist regarding culprit foods, and age of tolerance depending on the country of origin. We aimed at describing the characteristics of a French population of children with FPIES, and define risk factors for failure during challenge.

**Methods:**

Data from 179 children who were referred for FPIES in two pediatric tertiary centers between 2014 and 2020 were retrospectively collected. The diagnosis of FPIES was based on international consensus guidelines. Clinical characteristics, culprit food, and age at resolution were assessed. Tolerance was defined as no adverse reaction after OFC or accidental exposure.

**Results:**

In the 192 described FPIES, the age at first symptoms was 5.8 months old. The main offending foods were cow's milk (60.3%), hen's egg (16.2%), and fish (11.7%). Single FPIES was observed in 94.4% and multiple FPIES in 5.6% of cases. The age at resolution of FPIES was 2.2 years old, and resolution occurred later for fish than for milk (2.9 years vs. 2.0, *p* = 0.01). Severe acute FPIES was a risk factor for delayed resolution (RR: 3.3 [1.2–9.2]), but not IgE sensitization. Performing a food challenge within 12 months after the first reaction increased the risk of failure (OR: 2.6 [1.1–6.6]).

**Conclusion:**

In this French cohort of children with FPIES, the main culprit foods were ubiquitous. Rice, oat, and soy were rarely or not involved. Multiple FPIES was infrequent. Our data confirmed the overall good prognosis of FPIES, the later resolution of FPIES to fish and in the case of severe acute FPIES.

## 1 INTRODUCTION

Food protein‐induced enterocolitis syndrome (FPIES) is a non‐IgE‐mediated food allergy. Its incidence is estimated between 0.015% and 0.7%.^1^, ^2^, ^3^, ^4^ In the absence of biomarkers, the diagnosis of FPIES is based on clinical presentation. International diagnosis criteria have recently been proposed to improve its diagnosis.^5^ Acute FPIES is defined by typical repetitive vomiting starting 1–4 h after ingestion of the culprit food, in association with at least 3 minor criteria.^5^ These include some other episode of repetitive vomiting after eating the same or different foods, lethargy, pallor, the need for an emergency department visit or intravenous fluid support; diarrhea, hypotension, or hypothermia.^5^ Chronic FPIES occurs when the food is regularly consumed, and is mainly reported with cow's milk (CM) and soy formula ingestion. The diagnosis of chronic FPIES is based on the presence of intermittent emesis, chronic diarrhea, poor weight gain or failure to thrive, which improve after several days to weeks of exclusion of the offending food. After a period of avoidance, acute typical symptoms occur upon reexposure.^6^ Severe forms of acute FPIES may lead to dehydration, and hypovolemic shock is reported in 15%–33% of acute FPIES cases.^7^, ^8^, ^9^ IgE sensitization to the culprit food is unusual but may be observed in atypical FPIES.^5^ Although oral food challenges (OFCs) are not necessary for diagnosis when the typical symptoms are present, they are useful in doubtful cases to confirm the diagnosis.^5^ The offending foods depend on geographic origins.^6^ The most frequent culprit foods are CM in Europe and North America,^10^, ^11^, ^12^, ^13^, ^14^, ^15^, ^16^, ^17^ soya, rice, and grains in North America and Australia,^2^, ^4^, ^8^, ^16^, ^18^ and fish in Mediterranean countries.^11^, ^19^ Resolution of FPIES is expected by school age in the majority of cases.^6^ OFCs are performed to assess tolerance to the food in question, generally 12–18 months after the last reaction.^5^

In this study, we aimed to describe FPIES in a large population of French children for the first time, using international diagnosis criteria, to describe its natural history, and to define risk factors for failure during OFC.

## 2 METHODS

### 2.1 Subjects

Data from children with FPIES, referred consecutively to two French pediatric centers (Trousseau and Necker‐Enfants Malades, Assistance Publique – Hôpitaux de Paris) between January 2014 and April 2020 were retrospectively collected. The diagnosis of acute FPIES was *confirmed* if recurrent vomiting was associated with at least three minor criteria,^5^ or in the presence of typical vomiting after performance of an OFC.^5^, ^20^ The diagnosis of acute FPIES was *presumptive* when the recurrent vomiting was associated with only two minor criteria, in the absence of skin or respiratory symptoms, and without any argument for a differential diagnosis.^5^, ^6^ The diagnosis of chronic FPIES was *confirmed* in the presence of acute‐on‐chronic typical symptoms.^5^ The diagnosis of chronic FPIES was considered to be *presumptive* in the absence of any acute phase, in children with compatible symptoms, including chronic diarrhea, vomiting, with significant improvement within a few days after avoidance of the offending food, and after exclusion of differential diagnosis (food protein‐induced enteropathy, gastrointestinal reflux, cyclic vomiting, anatomical gastrointestinal obstruction, infectious gastroenteritis and inborn errors of metabolism).^5^, ^6^ When the diagnosis criteria of FPIES were lacking, children were excluded from the study.

### 2.2 Description of FPIES

Clinical data related to FPIES were collected: age at onset of first symptoms, age at diagnosis, culprit food(s), description of symptoms, age at OFC, age at acquisition of tolerance (defined as age at negative OFC or claimed regular consumption of the food in question without any reaction), and personal and familial first‐degree relative history of atopic disease. Atopic disorder was defined as a history of IgE‐mediated food allergy, allergic rhinoconjunctivitis, asthma or atopic dermatitis and/or a positive skin prick test (SPT) or specific IgE. SPTs were performed with the offending food using either a commercial allergen extract or as a prick‐by‐prick using fresh food or milk. The SPT was considered to be positive if the diameter of the wheal was at least 3 mm larger than the negative control (saline).^21^ Specific IgE values were considered to be positive if higher than 0.35 kU/L (ImmunoCap™, Thermo Fisher Scientific, Phadia AB, Uppsala, Sweden).^21^

*Multiple* FPIES was defined as FPIES to several groups of foods, as opposed to single FPIES. Several species of fish were considered as a unique food group, as were vegetables from the cucurbit family for example, *Solid foods* referred to food other than mammal's milk.

Acute FPIES was defined as *severe* if the patient had needed a rapid vascular filling and/or hospitalization due to dehydration or hypovolemic shock, persistent hypotonia or malaise.

*Persistent* FPIES was defined as FPIES without the acquisition of tolerance at the end of the follow‐up and after at least 1 year after the first symptoms.

### 2.3 Oral food challenges

OFCs were mainly performed to confirm or exclude the tolerance in patients previously diagnosed with FPIES, in medical day units. After 2017, OFC protocols were adapted from the international consensus. An appropriate age‐serving size was given in a single portion or in two to three equal doses administered over 30 min, with a peripheral intravenous access, followed by an observation period of 4 h^5^ This timing and the interval were let to the judgment of the allergist, depending on history of severe reaction and type of food, according to the international recommendations.^5^ Children were considered to be tolerant if no symptoms occurred within 4 h after ingestion of the food in question, and they were able to tolerate one age‐appropriate serving regularly at home. We took into account the successful reintroduction performed at home (accidental or voluntary exposure). An OFC failure was diagnosed in the case of recurrence of vomiting, even if isolated, as suggested by Leonard et al.^20^

### 2.4 Statistical analyses

Continuous values were expressed as median and interquartile range (IQR) values, or in raw values with a percentage. Statistical analyses and figures were performed using GraphPad Prism version 5.3 for Windows and R statistical analysis software. Mann–Whitney U‐tests were performed to compare non‐parametric variables. Spearman's coefficients were calculated to assess non‐parametric correlations. Proportions and risk factors were compared using Chi^2^ test or Fisher's exact test where appropriated. Multivariable logistic regression analysis was performed to determine their independent contributions to failure of first OFC (relative risk: RR and odds ratio: OR were expressed with the confidence interval 95%). A *p* < 0.05 was considered to be significant. Kaplan–Meier survival analyses were performed to estimate the likelihood of outgrowing FPIES by age. The non‐tolerant patients were censored at the age of the last follow‐up (OFC, consultation or last attempt at a phone call if contact lost, as a follow‐up).

### 2.5 Ethics

The study was approved by the French Pediatric Hepato‐Gastroenterology and Nutrition's Ethics Committee (no. 2020–023 of May 2020).

## 3 RESULTS

### 3.1 General characteristics of the population

**FIGURE 1:**
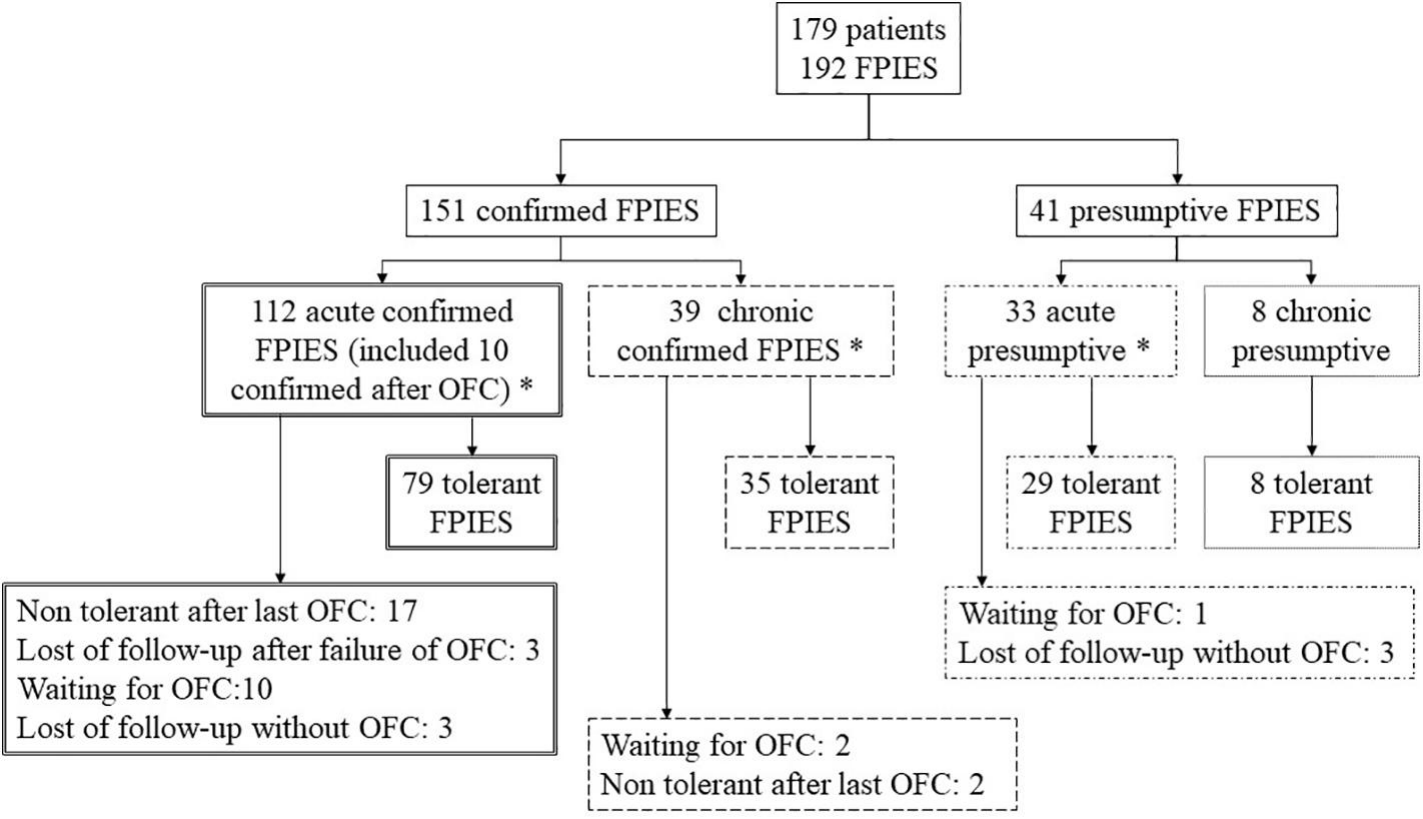
Flow chart. *Multiple FPIES *n* = 10: (1) acute confirmed form to soy and chronic confirmed to cow's milk; (2) acute confirmed form to maize and hen's egg and chronic confirmed to cow's milk; (3) acute confirmed form to cow's milk, chicken, hen's egg and green beans; (4) acute confirmed form to cow's milk and acute presumptive form to beef; (5) acute confirmed form to tomato and coconut; (6) acute presumptive form to hen's egg and rice; (7) acute confirmed form to beef and chronic confirmed form to cow's milk; (8) acute confirmed form to rice and banana; (9) acute confirmed form to cow's milk and raspberries; (10) acute confirmed form to avocado and cashew nuts

One hundred and seventy‐nine (*n* = 179) children with FPIES were included (Figure 1). The female to male ratio was 0.88 (53.1% of boys; Table 1). The median age at the onset of the first symptoms of FPIES was 5.8 months (3.0–8.0) and was younger for CM than for solid foods (Table 2). Personal and familial histories of atopic disease are presented in Table 1.

**TABLE 1:** General characteristics of the population

	Total population (*n* = 179)
Sex ratio (female/male) (% boys)	84/95 (53.1%)
Age at first symptoms (months)	5.8 (3.0–8.0)
Positive culprit food specific IgE	28/180 (14.7%)
Positive skin prick test for the culprit food	5/121 (4.1%)
Personal atopic history	67/165 (40.6%)
IgE‐mediated food allergy	10/179 (5.6%)
Asthma	22/164 (13.4%)
Atopic dermatitis	47/165 (28.5%)
Rhinoconjunctivitis	6/164 (3.7%)
First relative atopic history (self‐reported)	113/168 (67.3%)
Father	
IgE‐mediated food allergy	7/169 (4.1%)
Asthma	18/169 (10.7%)
Atopic dermatitis	18/169 (10.7%)
Rhinoconjunctivitis	30/169 (17.8%)
Mother	
IgE‐mediated food allergy	20/168 (11.9%)
Asthma	28/168 (16.7%)
Atopic dermatitis	24/168 (14.3%)
Rhinoconjunctivitis	47/168 (28.0%)
Siblings	
IgE‐mediated food allergy	9/42 (21.4%)
Asthma	9/42 (21.4%)
Atopic dermatitis	8/42 (19.1%)
Rhinoconjunctivitis	4/42 (9.5%)

**TABLE 2:** Comparisons between cow's milk and the other culprit foods, in terms of age at the onset of the first symptoms, first OFC, and resolution

	Cow's milk (*n* = 108)	Solid foods (*n* = 83)	Hen's egg (*n* = 29)	Fish (*n* = 21)	Vegetables, legumes and fruits (*n* = 21)	Rice (*n* = 6)	Meat (*n* = 5)
Age at first symptoms (months)	3.0 (1–5.3)	8.0 (6.0–12.0)*	8.0 (6.0–9.0)*	8.0 (6.0–12.5)*	10.5 (7.8–12.3)*	5.0 (4.3–5.8)	11.1 (9.5–14.8)**
	(*n* = 107)	(*n* = 76)	(*n* = 27)	(*n* = 19)	(*n* = 20)	(*n* = 6)	(*n* = 4)
Age at first OFC (years)	1.8 (1.3–2.5)	2.6 (1.9–3.3)*	1.9 (1.7–2.8)	3.0 (2.6–5.2)*	2.6 (2.1–3.1)**	1.9 (1.4–2.7)	5.3 (4.3–6.2)**
	(*n* = 105)	(*n* = 67)	(*n* = 26)	(*n* = 16)	(*n* = 17)	(*n* = 5)	(*n* = 3)
Tolerant patients	95 (88.0%)	56 (67.5%)	24 (82.8%)	11 (52.4%)	14 (66.7%)	5 (83.3%)	2 (40.0%)
Age of tolerance (years)	2.0 (1.5–2.9)	2.6 (1.9–3.0)***	2.2 (1.8–2.8)	2.9 (2.3–4.5)***	2.6 (2.0–3.1)	1.9 (1.4–2.7)	5.2 (4.2–6.1)
	(*n* = 95)	(*n* = 55)	(*n* = 24)	(*n* = 11)	(*n* = 13)	(*n* = 5)	(*n* = 2)

*Note*: Age expressed in median months or years (interquartile range). OFC: Oral Food Challenge. Comparison between cow's milk and the other foods, except for difference between tolerance for milk and meat (“Meat” group too low).

*p < 0.001; **p < 0.01; ***p < 0.05.

### 3.2 FPIES characteristics

A total of 192 FPIES cases were reported (Figure 1). The diagnosis of FPIES was *confirmed* in 151 cases and was *presumptive* in 41 cases (Table 3). Children with *confirmed* or *presumptive* FPIES did not differ in terms of sex ratio, atopic status, age at tolerance or tolerance rate (Table 3). Acute or recurrent chronic vomiting were present in all of the children.

**TABLE 3:** FPIES characteristics

	Confirmed (*N* = 151)	Presumptive (*N* = 41)	*p* value
Acute FPIES	112 (74.2%)	33 (80.5%)	0.5
Chronic FPIES	39 (25.8%)	8 (19.5%)	0.5
Sex ratio (male/female) (% boys)	76/75 (50.3%)	20/21 (48.8%)	1.0
Atopic history			
Personal	52/140 (37.1%)	20/37 (54.1%)	0.09
Paternal	44/145 (30.3%)	16/36 (44.4%)	0.1
Maternal	68/145 (46.9%)	19/36 (52.8%)	0.6
Siblings	17/33 (51.5%)	4/11 (36.4%)	0.5
Age at first symptoms (months)	5.0 (3.0–8.0)	6.0 (3.7–10.5)	**0.049**
Main culprit food			
Cow's milk	93 (61.6%)	15 (36.6%)	**0.005**
Hen's egg	20 (13.2%)	9 (22.0%)	0.2
Fish	13 (8.6%)^a^	8 (19.5%)^b^	0.9
Single fish	9/13	5/8	
Multiple fish	4/13	1/8	
Other foods	25 (16.6%)^c^	9 (22.0%)^d^	0.5
Minor criteria (acute FPIES) (mean)	3.7 per FPIES	2 per FPIES	**<0.001**
Recurrent vomiting after same food	98/112 (87.5%)	25/33 (75.8%)	0.1
Lethargy	90/112 (80.4%)	18/33 (54.5%)	**0.006**
Pallor	71/112 (63.4%)	6/32 (18.2%)	**<0.001**
Emergency department visit	49/112 (43.8%)	6/33 (18.2%)	**0.008**
Diarrhoea	41/112 (36.6%)	7/33 (21.2%)	0.1
Intravenous fluid support	39/112 (34.8%)	1/31 (3.2%)	**<0.001**
Vomiting after different food	25/112 (22.3%)	3/33 (9.1%)	0.1
Hypotension	6/87 (6.9%)	0/23	0.3
Hypothermia	0	0	–
Positive food specific IgE	20/142 (14.1%)	8/38 (21.1%)	0.3
Severe form (dehydration, hypovolemic shock, persistent hypotonia, or malaise)	13 (8.6%)	0	0.07
Age at first OFC (years)	2.0 (1.4–2.9)	2.3 (1.6–2.9)	0.3
Age at tolerance (years)	2.1 (1.7–3.0)	2.5 (1.6–2.9)	0.7
Number of tolerant patients	114 (75.5%)	37 (90.2%)	0.05

*Note*: The bold numbers indicates *p* < 0.05.

^a^ Confirmed FPIES to fish: codfish only *n* = 8, salmon only *n* = 1, hake and salmon *n* = 2; codfish and salmon *n* = 1; codfish and hoki *n* = 1.

^b^ Presumptive FPIES to fish: codfish *n* = 4; tuna *n* = 1; codfish + hoki + salmon + sole *n* = 1; nonspecified *n* = 2.

^c^ Confirmed FPIES to other foods: vegetables *n* = 6 (broccoli, cucurbits, green beans, mushroom, and sweet potato); legumes *n* = 2 (green peas, soy); fruits *n* = 8 (avocado, banana, cashew nuts, coconut pineapple, raspberry, and tomato); cereals *n* = 6 (rice, wheat); meat *n* = 3 (beef, chicken).

^d^ Presumptive FPIES to other foods: vegetables *n* = 2 (cucurbits, mushroom); legumes *n* = 1 (peanut); fruits *n* = 2 (apple, apricot); cereals *n* = 2 (rice); meat *n* = 2 (beef).

Children with acute FPIES had a median of 3.0 minor criteria (2.0–4.0; mean 3.3; maximum: 7). The most frequent minor criteria were recurrent episodes of repetitive vomiting after eating the same culprit food (84.8%), followed by lethargy (74.5%), pallor (53.1%), the need for an emergency department visit (37.9%), diarrhea (33.1%), the need for intravenous fluid support (27.6%), vomiting after eating a different food (19.3%), and hypotension (4.1%). Hypothermia was not recorded. Lethargy, pallor, an emergency department visit, and intravenous fluid support were more often found in confirmed FPIES cases (*p* < 0.01; Table 3).

13 children (7.3%) experienced severe confirmed acute FPIES (Table 3). Two patients required hospitalization in an intensive care unit owing to severe dehydration following ingestion of CM. Eleven patients needed rapid vascular filling during an OFC.

A total of 47 children (26.1%) had chronic FPIES, and CM was the only elicitor of chronic FPIES.

**FIGURE 2:**
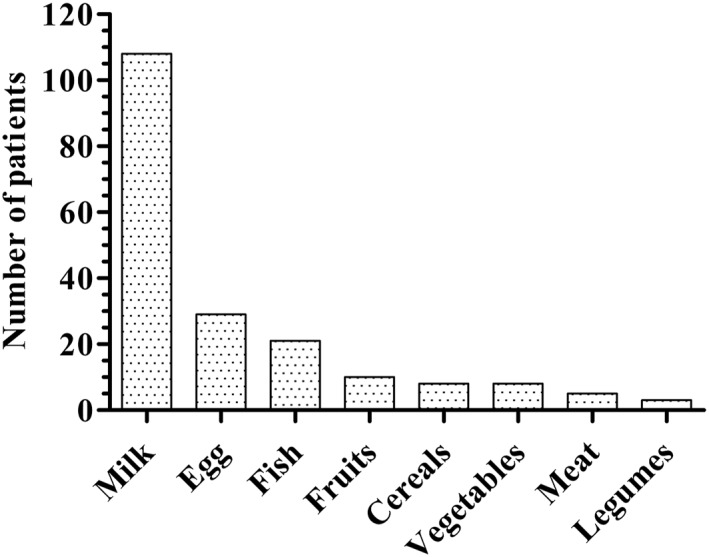
Repartition of patients according to the offending foods. **Milk:** *n* = 108; **Hen's egg:** *n* = 29; **Fish:** *n* = 21; **Fruits:** *n* = 10 (apple, apricot, avocado, banana, cashew nuts, and coconut (*n* = 2), pineapple, raspberry, and tomato); **Cereals**: *n* = 8 (maize, rice and rice hydrolysate (*n* = 6), wheat); **Vegetables:** *n* = 8 (broccoli, cucurbits (*n* = 2), green beans, mushroom (*n* = 2), sweet potato (*n* = 2)); **Meat:** *n* = 5 (beef (*n* = 3), chicken (*n* = 2)); **Legumes:** *n* = 3 (green peas, peanut, and soy). Total: 179 patients included 10 patients with multiple FPIES, that is 192 FPIES

One hundred and sixty‐nine (94.4%) children had single FPIES, and 10 (5.6%) had multiple FPIES. Twenty‐three culprit foods were identified. CM was involved in 108 children (60.3%), hen's egg in 29 (16.2%), and fish in 21 (11.7%; Figure 2). Among the 10 multiple FPIES cases reported, CM was involved in six cases. One child had FPIES to 4 foods (CM, chicken, hen's egg, and green beans), another one to three foods (CM, hen's egg, and maize), and eight to two foods: CM and beef/veal (*n* = 2), CM and soy, CM and raspberry, rice and hen's egg, rice and banana, coconut and tomato, avocado and cashew nuts.

### 3.3 IgE sensitization

IgE sensitization to the culprit food at any moment was found in 28/180 FPIES (14.7%). SPTs with the offending food were performed in 121/192 cases (63.0%) and were positive in five cases (4.1%; Table 1). One child developed an IgE‐mediated allergy with the culprit food over time: she had a confirmed typical FPIES to CM until the age of 3 without any sensitization, and had thereafter developed an immediate urticaria and rhinoconjunctivitis after ingestion of CM at 5 years old, with a positive SPT and increased CM's IgE: 10.9 kU/L. By contrast, a child with a history of IgE‐mediated allergy to CM during the first year of life (urticaria after cow's milk ingestion and specific CM's IgE: 4.9 kU/L at the age of 1 month), switched to FPIES to CM after 9 months of age. Her specific IgE was negative at this time, and she had repetitive vomiting, without skin or respiratory symptoms, during an OFC to CM at the age of 10 months.

### 3.4 OFC

Two hundred and twelve (*n* = 212) OFCs were performed to assess tolerance. A first OFC was performed in 173 children at 2.0 years of age (1.5–2.9), with a success rate of 74.6%. A second OFC was performed in 33 children at 2.3 years of age (2.0–3.5), with a success rate of 57.6%. A third OFC was performed in six children at 3.1 years of age (2.6–3.8), with a success rate of 100%. The interval between two OFCs was 11.7 months (7.0–15.8). In 19 cases of FPIES (12 with confirmed acute FPIES, 2 with confirmed chronic FPIES, 4 with presumptive acute FPIES, and 1 with chronic FPIES), patients reintroduced the food on their own, without any reaction, at a median age of 2.7 years (2.2–3.3).

For milk, the first OFC was performed at the median age of 1.8 years, which was earlier than for other foods (*p* < 0.001), fish (*p* < 0.001), meat and vegetables/legumes/fruits (*p* < 0.01), but not different from hen's egg and rice (Table 2).

Nineteen OFCs were still not performed, because patients were too young and/or their last reaction was too recent and/or because of family refusal. Nine children were lost in the follow‐up, including six without the performance of any OFCs, and three after one attempt at an OFC. Five out of these nine lost patients had FPIES to fish.

### 3.5 Evolution and risk factor of failure of OFCs or prolonged FPIES

**FIGURE 3:**
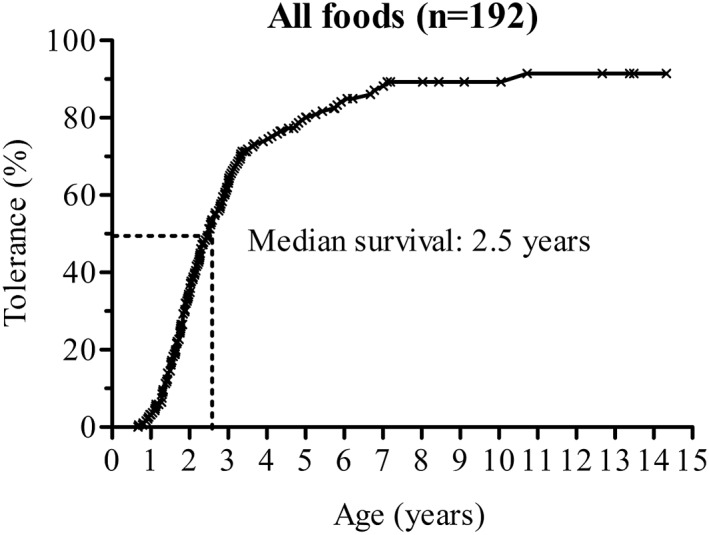
Overall Kaplan–Meier survival curve

**FIGURE 4:**
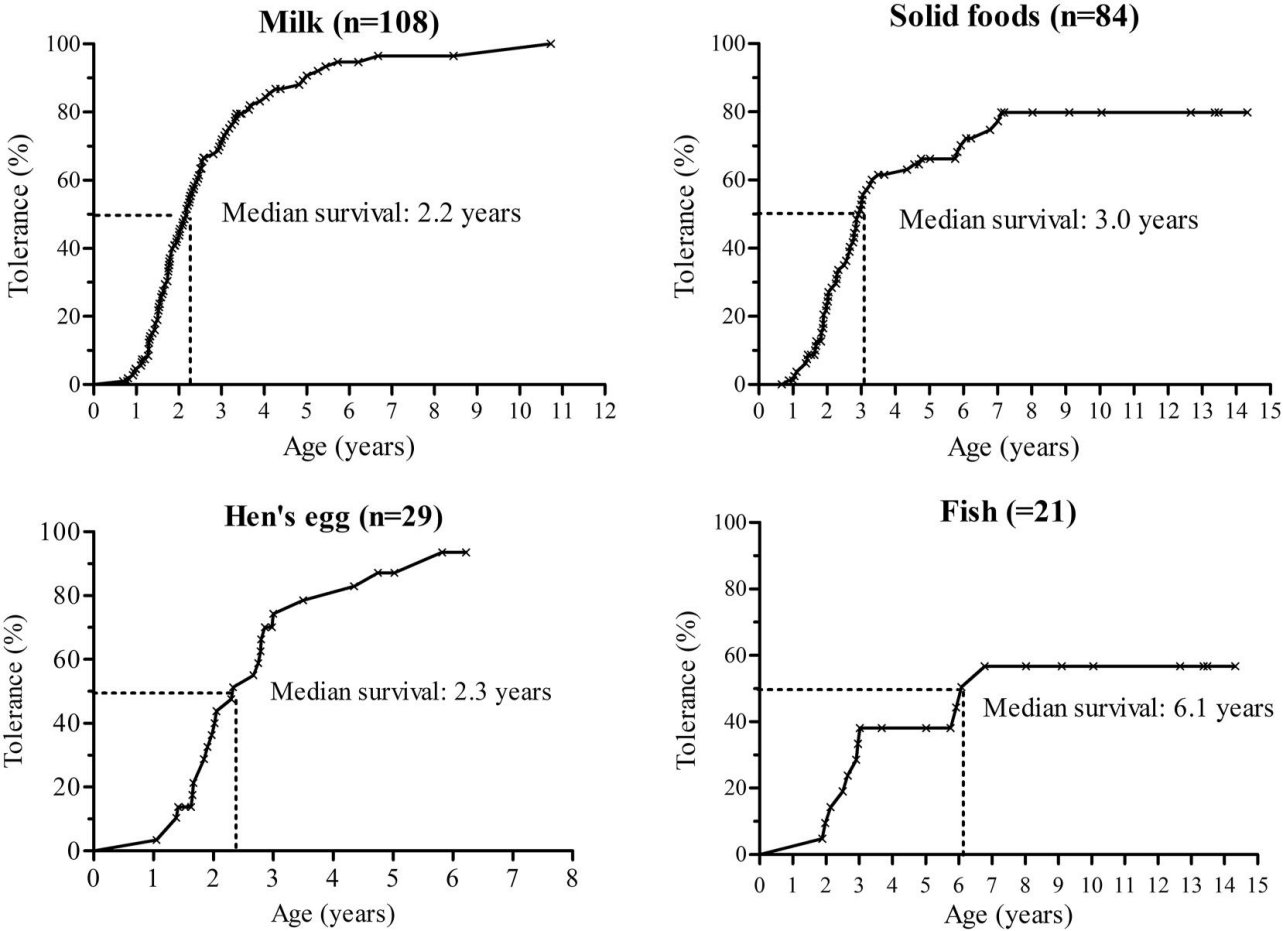
Kaplan–Meier survival curves for milk, solid food, hen's egg, fish and likelihood of FPIES resolution by age and food. **Milk:** likelihood of FPIES resolution by 1 year of age: 5.6%; by 2 years of age: 42.8%; by 3 years of age: 70.9%; by 5 years of age: 90.7%; **Solid foods**: likelihood of FPIES resolution by 1 year of age: 2.5%; by 2 years of age: 24.4%; by 3 years of age: 52.7%; by 5 years of age: 66.2%; **Hen's egg**: likelihood of FPIES resolution by 1 year of age: 3.5%; by 2 years of age: 40.0%; by 3 years of age: 74.3%; by 5 years of age: 87.1%; **Fish**: likelihood of FPIES resolution by 1 year of age: 4.7%; by 2 years of age: 14.3%; by 5 years of age: 38.1%; by 10 years of age: 56.7%

The median age at the last review of medical records was 2.3 years old (1.7–3.3, maximum 14.3). At this time, 151 out of 192 culprit foods were successfully reintroduced (78.6%). Eighty‐eight percent of children with FPIES to CM were tolerant, as were 82.8% of those reactive to hen's eggs, and 52.4% to fish (Table 2). Among the tolerant patients, the overall age of tolerance was 2.2 years of age (1.7–3.0, *n* = 150). Kaplan–Meier curves showed an overall median survival of FPIES at 2.5 years of age, with a global resolution rate of 80.1% at 5 years of age (Figure 3). The resolution rate at 5 years of age was higher for FPIES to CM than to fish, and was similar for FPIES to CM and hen's egg (Figure 4).

Performing a reintroduction within the 12 months after the onset of FPIES was associated with an increased risk of failure of the first OFC (OR: 2.6 [1.1–6.6], *p* = 0.04 in multivariate analysis), particularly in children with a severe form of FPIES (OR: 40 [4.8–333.3], *p* < 0.001; Table 4).

**TABLE 4:** Outcomes of first OFC according to different factors

Univariate analysis				
	Success (*n* = 129)	Failure (*n* = 44)	RR [IC95%]	*p* value
Age at first symptoms (years)	0.4 [0.3–0.7]	0.4 [0.1–0.7]		0.4
Age at first OFC (years)	2.1 [1.6–3.0]	1.7 [1.2–2.7]		0.6
Delay between first symptoms and first OFC	1.5 [1.1–2.3]	1.3 [0.9; 2.1]		0.7
OFC performed in the first year after first symptoms	23/128 (17.9%)	15/43 (34.8%)	1.3 [1.0–1.7]	**0.02**
Age at tolerance	2.1 [1.6–3.0]	2.3 [1.9–3.8]		0.1
History of severe reaction(s)	1 (0.8%)	10 (22.7%)	4.3 [3.0–6.2]	**<0.001**
Positive culprit food specific IgE	19/122 (15.6%)	9/42 (21.4%)		0.4
Chronic FPIES	34/118 (28.8%)	11/42 (26.2%)		0.8
Multiple FPIES	11 (8.5%)	4 (9.1%)		1
Personal atopic history	53/120 (44.2%)	16/42 (38.1%)		0.5
Familial atopic history	80/121 (66.1%)	27/43 (62.7%)		0.7

Multivariate analysis		
	OR [IC95%]	*p* value
OFC Performed in the first year after first symptoms	2.6 [1.1–6.6]	**0.04**
History of severe reaction(s)	40 [4.8–333.3]	**<0.001**

*Note*: The bold numbers indicates *p* < 0.05.

Severe acute reactions increased the risk of persistent FPIES (RR: 3.3 [1.2–9.2], *p* = 0.03). Six patients with a history of severe reactions out of 13 (46.2%) were not tolerant after a median duration of up to 4.3 years of age. The seven other patients were tolerant at 2.3 years of age.

IgE sensitization against the culprit food was not associated with a longer duration of FPIES among tolerant patients (*p* = 0.3) and was not a risk factor of failure of OFC (*p* = 0.4).

Neither personal nor familial atopic history were risk factors of persistent FPIES (*p* = 0.15 and 0.9, respectively).

## 4 DISCUSSION

In this study, we described the characteristics of a large population of 179 French children with FPIES according to international guidelines. We found that (i) culprit foods were ubiquitous as in other international cohorts, but some specific characteristics existed, (ii) persistent FPIES was more frequent for fish than for other foods, and in case of severe acute FPIES, but IgE sensitization was not associated with longer duration of FPIES, (iii) performing OFC within 12 months after the first reaction increased the risk of failure.

In our study, the main culprit food was CM, followed by hen's egg, and fish, which differs from the findings in other countries.^11^, ^15^, ^17^, ^22^, ^23^, ^24^, ^25^, ^26^, ^27^, ^28^, ^29^ The most frequent culprit food was fish (54%) in Greece^19^ and Spain (70.6%),^27^, ^30^ rice in Australia^31^, ^32^, ^33^ and the USA,^18^ and oats (34.5%) in Taiwan.^34^ Soy is frequently reported as a trigger food by North American, British, Australian and Israeli cohorts^4^, ^8^, ^10^, ^16^, ^33^, ^35^, ^36^ and was infrequent in our population. Food habits, geographic origins, genetic factors, microbiota, and other environmental pre‐ or postnatal factors may explain these differences.^1^, ^10^, ^37^

Among the 108 patients with FPIES to CM, only 2 had a documented FPIES to beef or veal. One patient had single FPIES to beef. Cross‐reactivity between CM and beef is estimated at up to 20% in IgE‐mediated allergies.^38^ This meat is frequently avoided by caregivers of FPIES‐children.^9^ However, the prevalence of FPIES to beef is estimated between 0.8% and 3.0% of children with FPIES.^2^, ^4^, ^8^, ^10^, ^18^, ^25^, ^29^ Although beef is considered as a “moderate‐risk” food,^20^ our data suggest that having FPIES to CM does not increase the risk of associated FPIES to beef.^33^

The overall age of resolution of FPIES was 2.2 years of age for all foods. The age at resolution was based on the day of performance of an OFC and thus may be overestimated.^6^ Some data suggests that tolerance occurred later for solid foods than for CM, but results diverge.^4^, ^13^, ^16^ Miceli Sopo et al.^13^ reported an age of tolerance of 2.0 years for FPIES to CM and 4.4 years for other foods (*p* < 0.0006), whereas other authors did not find any difference.^4^, ^16^ We found that the acquisition of tolerance was delayed by 6 months for solid foods compared to CM. Previous studies suggested that the later age of tolerance relates to the ingestion of seafood products^6^, ^11^, ^33^, ^39^ and may occur more frequently in cases of multiple FPIES.^6^ Resolution of FPIES to fish is around 18.8%–57.0% of cases between 3 and 4.5 years of age.^19^, ^32^, ^39^, ^40^, ^41^ We found a similar rate of 38% tolerance at 4.0 years of age, with an older age of resolution for fish than CM. Due to the low prevalence of multiple FPIES in our cohort, we were unable to compare the age of resolution of single and multiple FPIES.

The recurrence of repetitive vomiting, lethargy and pallor were the three most frequently observed minor criteria. Lethargy and pallor are criteria with large variability in studies (from 3.8%^19^ to 100%^17^ for lethargy; from 14%^1^ to 98.7%^19^ for pallor). We did not find any hypothermia in FPIES histories, as is the case for Dieme et al.^26^ Hypothermia is indeed an uncommon symptom, from 2% in Sweden^29^ to 10% in Australia,^2^, ^31^ but up to 31.2% of patients according to caregivers from the International FPIES Association.^9^ Some minor criteria (such as hypothermia, hypotension, pallor, and lethargy) are difficult to identify during the in‐depth family interviews, and even worse in retrospective reviews of medical records.^30^

We included patients suffering from acute and chronic presumptive FPIES if the history was compatible with the diagnosis of FPIES without an argument for a differential diagnosis, as previously described.^5^, ^6^ The hypothesis that this may affect our results is unlikely because general characteristics and the prognosis in children with confirmed and presumptive FPIES did not differ. Recent data demonstrated how the different FPIES diagnostic criteria proposed over time provide conflicting results in patients with a high clinical suspected likelihood of acute FPIES.^30^ Despite multiple reactions to the same offending food, one quarter of the cohort of Vazquez‐Ortiz et al.^30^ did not meet the criteria from the “2017 consensus,”^5^ especially when severity was mild,^30^ as was the case for us. Accordingly, we cross‐referenced our 145 acute FPIES patients to other definitions. We found 61.4% of patients who fulfilled the Powell criteria modified by Sicherer/1998,^7^ 61.4% (up to 84.8% without the age criteria) according to Leonard/2012,^42^ 24.1% with Miceli Sopo's 2013 definition (up to 27.6% without the age criteria),^43^ 91.1% according to Lee/2017.^32^ Different phenotypes of FPIES may exist depending on geographic origins or culprit foods which could explain the variability of the symptoms previously described.^30^

Performing an OFC in the first year after the diagnosis resulted in an increased risk of failure, confirming that an OFC should generally be considered at least 12 months after the last reaction.^6^ For fish, one must be even more patient, because experts recommend postponing the performance of an OFC until 5 years of age or older,^6^ and testing tolerance to alternative fish to avoid an unnecessarily fish‐free diet.^39^ Like Infante et al.,^39^ we found that severe reactions at any moment were associated with a risk of longer duration of FPIES.

Limited data suggest that atypical FPIES with positive specific IgE is associated with delayed tolerance.^6^, ^8^, ^16^ This was not confirmed in our study population, like in the recent Swedish^29^ and Greek cohorts,^44^ although sensitization (IgE and/or SPT; 14.7%) were similar compared to other studies (11.1%–34%).^10^, ^11^, ^18^, ^22^, ^25^, ^26^ Atopic disorder was found in 41% of patients and eczema in 29%. This is concordant with American and Australian cohorts where eczema is reported in 11%–57% of patients with FPIES.^5^ Children with FPIES often have associated atopic conditions (atopic dermatitis, IgE‐food allergy, asthma, and allergic rhinitis).^4^ Even if FPIES is not an atopic disease *per se*, this suggests that FPIES and other atopic comorbidities share common pathophysiology.^45^, ^46^

We reported a lower frequency of multiple FPIES (5.6%) than in the literature which is commonly reported at around 30%.^2^, ^8^, ^10^, ^12^, ^15^, ^22^, ^26^, ^33^ This may result from the use of stringent criteria for the diagnosis of FPIES and the retrospective design of the study. Despite medical charts studied, for multiple FPIES in 13.4% of cases as per other series,^13^, ^17^, ^25^, ^27^, ^29^, ^30^, ^31^ we only retained FPIES with a specific clinical description. The prevalence of multiple FPIES ranges from 5.1%^19^ to 69.0%.^18^ These variations of prevalence could be explained by the fact that patients had been referred to tertiary centers in the case of multiple and more complex cases of FPIES. Secondly, it may be easier to diagnose multiple FPIES in children with a previous diagnosis of FPIES.^5^ It is interesting to note that, even if the incidence of single FPIES is generally more prevalent than multiple FPIES, families report in 69.7% of cases an avoidance of at least two food groups.^9^ Consequently, the risk of developing food aversion is significantly increased in FPIES triggered by three or more foods, by a factor of 3.^34^ Therefore, avoidance should be limited only to the confirmed offending foods. Supervised introduction allows for the prevention of unnecessary exclusion^20^ and overdiagnosis of multiple FPIES.

Our study had certain limitations. The decision to include patients with acute vomiting and only two minor criteria could be one such limit, as previously explained. The retrospective aspect of our study is another limitation, owing to missing data, and in particular in terms of the description of minor criteria and multiple FPIES. Familial history of atopic disease was self‐reported, which leads to a typical bias of over‐reporting allergic symptoms.^47^ In terms of further studies, researching a link between maternal feeding, mode of delivery, previous anti‐acid treatment and frequency of antibiotic use and the occurrence of FPIES could be interesting, by exploring the field of gut dysbiosis.

## 5 CONCLUSION

In summary, we reviewed a large French cohort of children with FPIES. The main culprit foods were CM, hen's egg, and fish. The overall prognosis remained good, as half of the cohort had outgrown FPIES by 2 years of age. FPIES to seafood products and severe forms of FPIES were associated with delayed tolerance. IgE sensitization was not a risk factor for persistent FPIES.

## CONFLICT OF INTEREST

None.

## AUTHOR CONTRIBUTION

1. Lemoine Anaïs, Colas Anne‐Sophie, Le Sébastien, Delacourt Christophe, Tounian Patrick, Lezmi Guillaume:
2. Have made substantial contributions to conception and design, or acquisition of data, or analysis and interpretation of data; and
3. Been involved in drafting the manuscript or revising it critically for important intellectual content; and
4. Given final approval of the version to be published. Each author should have participated sufficiently in the work to take public responsibility for appropriate portions of the content; and
5. Agreed to be accountable for all aspects of the work in ensuring that questions related to the accuracy or integrity of any part of the work are appropriately investigated and resolved.
